# Impact of Air Pollution on Age and Gender Related Increase in Cough Reflex Sensitivity of Healthy Children in Slovakia

**DOI:** 10.3389/fphys.2016.00054

**Published:** 2016-02-23

**Authors:** Silvia Demoulin-Alexikova, Jana Plevkova, Lenka Mazurova, Tomas Zatko, Mikulas Alexik, Jan Hanacek, Milos Tatar

**Affiliations:** ^1^Department of Pathophysiology, Jessenius Faculty of Medicine in Martin, Comenius University in BratislavaBratislava, Slovakia; ^2^Service d'Explorations Fonctionnelles Pédiatriques, Hôpital d'Enfants, Centre Hospitalier Universitaire de NancyVandœuvre-lès-Nancy, France; ^3^EA 3450 DevAH – Laboratoire de Physiologie, Faculté de Médecine, Université de LorraineVandœuvre-lès-Nancy, France; ^4^Department of Ophthalmology, Faculty Hospital of ŽilinaŽilina, Slovakia

**Keywords:** cough reflex, air pollutants, environmental exposure, environmental tobacco smoke, PM10, age differences, gender differences

## Abstract

**Background:** Numerous studies show higher cough reflex sensitivity (CRS) and cough outcomes in children compared to adults and in females compared to males. Despite close link that exists between cough and environment the potential influence of environmental air pollution on age- and gender -related differences in cough has not been studied yet.

**Purpose:** The purpose of our study was to analyse whether the effects of exposure to environmental tobacco smoke (ETS) from parental smoking and PM10 from living in urban area are implied in age- and gender-related differences in cough outcomes of healthy, non-asthmatic children. Assessment of CRS using capsaicin and incidence of dry and wet cough was performed in 290 children (mean age 13.3 ± 2.6 years (138 females/152 males).

**Results:** CRS was significantly higher in girls exposed to ETS [22.3 μmol/l (9.8–50.2 μmol/l)] compared to not exposed girls [79.9 μmol/l (56.4–112.2 μmol/l), *p* = 0.02] as well as compared to exposed boys [121.4 μmol/l (58.2–253.1 μmol/l), *p* = 0.01]. Incidence of dry cough lasting more than 3 weeks was significantly higher in exposed compared to not exposed girls. CRS was significantly higher in school-aged girls living in urban area [22.0 μmol/l (10.6–45.6 μmol/l)] compared to school-aged girls living in rural area [215.9 μmol/l (87.3–533.4 μmol/l); *p* = 0.003], as well as compared to teenage girls living in urban area [108.8 μmol/l (68.7–172.9 μmol/l); *p* = 0.007]. No CRS differences were found between urban and rural boys when controlled for age group. No CRS differences were found between school-aged and teenage boys when controlled for living area.

**Conclusions:** Our results have shown that the effect of ETS on CRS was gender specific, linked to female gender and the effect of PM10 on CRS was both gender and age specific, related to female gender and school-age. We suggest that age and gender related differences in incidence of cough and CRS might be, at least partially, ascribed to the effect of environmental pollutants. The role of age and gender in the effect of air pollution on cough strongly suggest some interplay of development with biological and behavioral factors.

## Introduction

Cough is the most frequent respiratory symptom that leads adults and children to use over-the-counter medications or seek medical help. Whether caused by various conditions, from serious to minor ones, it may considerably diminish quality of life of an individual and affect social interactions.

Numerous studies show that physiology, physiopathology and outcome measures of cough differ in many ways throughout childhood (Thach, 2001; Ioan et al., 2014), between children and adults (Chang, 2005, 2010), as well as between males and females (Fujimura, 1996; Fujimura et al., 1996; Dicpinigaitis and Rauf, 1998; Dicpinigaitis et al., 2001; French et al., 2004; Birring and Pavord, 2009; Kelsall et al., 2009; Lamprecht et al., 2013).

According to observations from delivery room, cough seems to be absent at birth and is only rarely present in healthy newborns. Cough starts to appear after 2nd month of life when child looses the protection of maternally acquired antibodies and becomes susceptible to upper respiratory infections (Thach, 2001, 2007). Later, it becomes the most frequent respiratory symptom in toddlers and preschoolers. Objectively measured incidence of cough in healthy subjects seems to decrease from school-age (1–34 coughs/24 h) (Munyard and Bush, 1996) to adulthood (0–16 coughs/24 h) (Hsu et al., 1994) and similar results can be found in studies using capsaicin cough sensitivity testing. According to them, cough reflex sensitivity (CRS) to capsaicin in young children decreases with age (Chang et al., 1996, 1997a,b) and toward adulthood by gender (Chang et al., 1997c,d; Kastelik et al., 2002; Varechova et al., 2008).

Concerning cough it seems that gender differences are age-related indeed. Surveys in adult patients with chronic cough attending specialist clinics have consistently showed a higher number of women (Irwin et al., 1998; Kastelik et al., 2002; Kelsall et al., 2009), with mean ratio of female to male patients with chronic cough is 2.1 (95% CI, 1.6–2.4) (Fujimura, 1996). Other studies have shown that CRS to inhaled tussigenic stimuli such as citric acid, tartaric acid and capsaicin is higher in women, both healthy volunteers and patients with chronic cough (Fujimura et al., 1990, 1996; Dicpinigaitis et al., 2001; Kastelik et al., 2002). Here, as suggested by Patberg (Patberg, 2011) the influence of estrogen on TRPV1 may predispose females to cough hypersensitivity. On the other hand, a recent worldwide survey of chronic cough has shown that two thirds of the patients attending specialist cough clinics were females (mean age 55 years) and the most common age of cough presentation was 60–69 years (Morice et al., 2014). Studying functional brain activity in response to capsaicin inhalation in young male and female healthy volunteers has shown significantly larger somatosensory response in females compared to males, despite the lower dose of capsaicin used in females (Morice et al., 2014), suggesting a sex related differences in central processing of cough.

However, higher cough outcomes in children compared to adults and in females compared to males may have, at least partially, physiology-based reasons. These could originate from age- and gender-related differences not only in anatomy and physiology of respiratory, immune or nervous system, but also in behavioral and socio-cultural factors that considerably affect respiratory health (Becklake and Kauffmann, 1999). The effect of those factors on cough physiology and physiopathology may be the result of interplay between organism and the state of its environment. Cough reflex, as an innate inbuilt defense mechanism serves an important protective role in the respiratory system by expelling foreign particles and irritants inhaled from surrounding environment. Bronchopulmonary vagal afferents mediating cough are activated not only by endogenous mediators released during inflammation or tissue injury but also by a wide range of mechanical and chemical irritants contained in our alimentation (capsaicin, mustard oil, wasabi) and outdoor or indoor environment (vehicle exhaust, cigarette smoke, particulate matter) (Grace et al., 2013). Exposition to these factors may provoke coughing by direct stimulation of afferents but it may cause structural or functional changes in neural pathways responsible for regulating cough, leading to sensitization of the cough reflex. In this case, protective role of the reflex is lost and exaggerated coughing occurs in response to stimuli that are otherwise sub-threshold to provoke cough (Mazzone and Canning, 2002). Numerous animal studies advocate that such sensitization of cough reflex is present in response to exposure to cigarette smoke, inflammation and allergens (Undem et al., 1999, 2002; Carr and Undem, 2001) and clinical observations suggests that unexplained chronic cough might be a long lasting consequence of upper respiratory tract infection or other noxious agents in susceptible subjects (Cook and Strachan, 1997; Jaakkola and Jaakkola, 2002; McGarvey, 2005). Indeed, a hypothesis of neuropathic origin of chronic cough have been raised recently (Chung et al., 2013) highlighting the possible link between airway exposure to infectious, irritant or allergic insults and vagal nerve injury resulting in cough hypersensitivity and hence, chronic cough.

Despite close link that exists between cough and environment, the potential influence of environmental air pollution on increased capsaicin cough sensitivity and higher cough outcomes in children compared to adults and in females compared to males has not been studied yet. The aim of our study was to analyse whether the effects of indoor air pollution related to environmental tobacco smoke (ETS) and outdoor air pollution related to living in urban area are implied in age- and gender-related differences in cough outcomes of otherwise healthy children. As increase of CRS to tussigenic agents is supposed to reflect sensitization of the neural circuits regulating this defensive reflex (Mazzone and Canning, 2002), it is hypothesized here that exposure to ETS and living in urban area both result in increased CRS and exposure to air pollutants participate in age- and gender-related changes of CRS, found in numerous studies cited above. Further, it is hypothesized that increase in CRS due to environmental exposures is translated to increased incidence of chronic cough, evaluated by parent completed questionnaire on respiratory health.

## Materials and methods

### Study area

Study was performed in years 2007-2008 in Turiec region in the northwest part of Slovakia. Measurements were performed at one elementary school and one secondary school in Martin, a town in the center of the region with 60,000 inhabitants, and in two elementary schools at countryside, in two villages near Martin in the distance of 4, 5, and 11 km (Kostany nad Turcom and Bela-Dulice). The results of environmental pollution report, measured in the center of Martin showed that the only source of important pollution is PM_10_ (the mass of particles with an aerodynamic diameter of ≤ 10 μm), with mean concentration of 38.4 μg/m^3^ over 1 year (Republic, 2007). However, maximal concentration limit for PM10 (50 μg/m^3^ over 24 h that cannot be exceeded more than 35x per month) has been exceeded several times. There is no available information on air pollution at countryside.

### Subjects

The inclusion criteria to enter the study were: no history of asthma, no history of allergic diseases, no symptoms of acute respiratory infection in the preceding 2 weeks before testing, no history of other diseases that could modulate the CRS (e.g., diabetes mellitus, gastroesophageal reflux disease). The study was approved by the Ethics Committee of Jessenius Faculty of Medicine Comenius University in Martin, Slovakia while the children's parents signed informed consent.

Overall, 290 healthy, non-asthmatic children volunteers (age range 8–17 years, mean age 13.3 ± 2.6 years, 138 girls/152 boys) were included into study. For further analysis, children were divided in two groups according to their age: school-aged children, aged 7–12 years (*n* = 103; 48 girls/55 boys) and teenage children, aged 13–17 years (*n* = 187; 90 girls/97 boys).

### Measurements

#### Spirometry

All subjects underwent initial screening of their basic lung functions measured by spirometry before and after capsaicin challenge (KoKo DigiDoser-Spirometer; nSpire health Inc., Louisville, CO, USA) to rule out airway obstruction.

#### Cough sensitivity testing

CRS was assessed using capsaicin cough challenge, performed in agreement with the ERS guidelines (Morice et al., 2007) with modification for pediatric use (we used a compressed air-driven nebuliser (model 646; DeVilbiss Health Care, Inc., Somerset, PA, USA) controlled by a dosimeter (KoKo DigiDoser-Spirometer; nSpire health Inc., Louisville, CO, USA) with an inspiratory flow regulator valve added (RIFR; nSpire health Inc., Louisville, CO, USA) to assign identical inspiratory flow rate during capsaicin inhalations in all subjects. Each subject inhaled saline randomly interposed among 12 inhalations of incremental capsaicin aerosol concentrations (0.61–1250 μmol/l). Each administration of saline and capsaicin aerosol was performed at 1 min intervals with the inhalation time set at 400 msec. The number of coughs within 30 s after aerosol administration was counted by two independent observers. The end-point of cough challenge was the inhalation of capsaicin concentration that provoked at least 5 coughs (C5) or when the maximum concentration of capsaicin (1250 μmol/l) was achieved. The concentration of capsaicin causing at least two coughs was assigned as CRS and concentration of capsaicin causing at least 5 cough was assigned as C5. For children that did not cough at any concentration of capsaicin, CRS value was assigned 1250 μmol/l.

### Parent completed questionnaire on respiratory health and environmental expositions

Information on both parents smoking habits and place of living (urban or rural area) were obtained by parent completed questionnaire. According to this, children were assigned as living in urban or rural area and as exposed (ETS) or not (no-ETS) to ETS. The presence of “wet” and “dry” cough lasting more than 3 weeks in the past year was also obtained by parent completed questionnaire. Cough was defined wet, if it was associated with cold or flu and dry if it wasn't associated with cold or flu (Faniran et al., 1999).

### Statistical analysis

Analysis was performed using SYSTAT12 software.

Values of CRS and C5 were log10 transformed as the data were skewed. Values of C5 remained skewed after logarithmic transformation and further factorial ANOVA analysis using C5 values could not be realized. CRS values are expressed as geometric mean (95% confidence interval). *P* < 0.05 was regarded as statistically significant.

Two factorial ANOVA design was used to test the effect of age group (school aged children vs. teenage children) and gender (female vs. male) on CRS. Three factorial ANOVA designs were used to test the effect of exposition to ETS on CRS, while controlling for age group and gender. This led to a 2 × 2 × 2 between group design: age group (school aged children vs. teenage children by gender (female vs. male) by exposition to ETS (ETS vs. no-ETS).

Three factorial ANOVA design was also used to test the effect of living area on CRS, while controlling for age group and gender. This led to a 2 × 2 × 2 between groups design: age group (school aged children (7–12 years) vs. teenage children (13–17 years) by gender (female vs. male) by living area (urban vs. rural). In the case of significant 2-way or 3-way interaction the multiple comparisons testing (Tukey) were used to analyse the nature of the factor-level effects. *P* < 0.05 was regarded as statistically significant. The frequencies of cough parameters were compared between different groups using Chi square test or Fisher Exact test, in the case of small sample size.

## Results

### Cough threshold data

#### Effect of age group and gender

Two factorial ANOVA design have shown a significantly higher CRS in school-age compared to teenage children, respectively [45.3 μmol/l (32.6–64.6 μmol/l) vs. 105.4 (81.1–137.1 μmol/l), *p* = 0.0005]. No significant difference in CRS was seen between girls and boys [65.6 μmol/l (47.5–90.4 μmol/l) vs. 72.9 μmol/l (53.9–98.6 μmol/l), *p* = 0.6]. No significant two way interaction between age group and gender was seen [*F*_(1, 286)_ = 0.115, *p* = 0.7].

#### Effect of ETS

Three factorial ANOVA design between “age group” by “gender” by “ETS” did not reveal a significant three way interaction. On the other hand, a significant 2-way interaction between gender and ETS was seen [*F*_(1, 282)_ = 7.102, *p* = 0.008], showing that the effect of ETS on CRS differs between two genders. The main effect of age group was significant [*F*_(1, 282)_ = 7.073, *p* = 0.008] and showed a significant difference in CRS between school aged and teenage children [40.7 μmol/l (26.8–61.8 μmol/l) vs. 91.9 μmol/l (59.8–141.2 μmol/l)].

**Figure 1:**
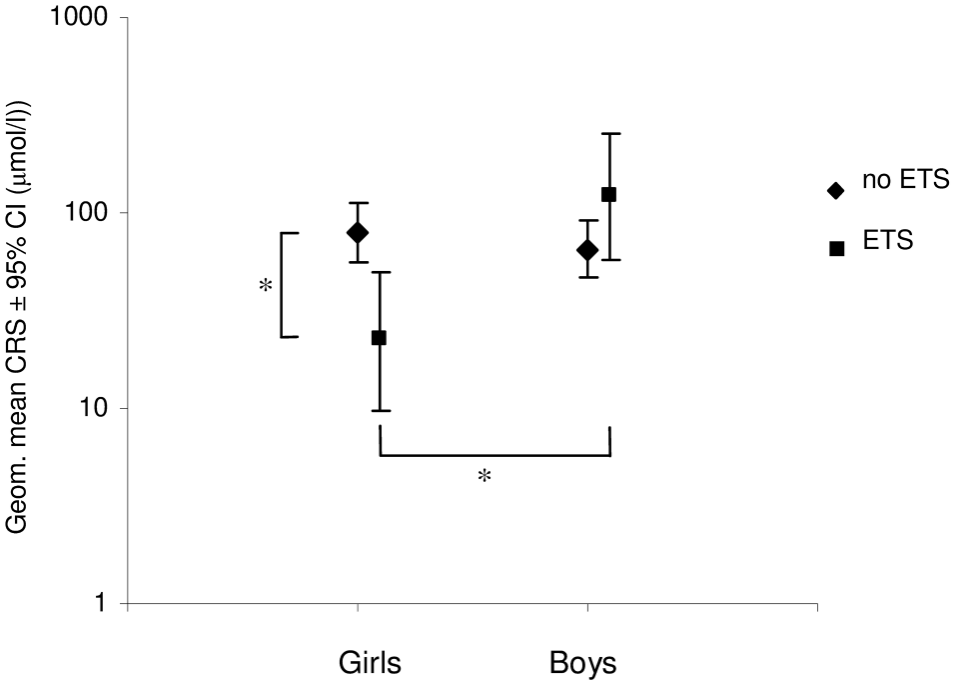
Impact of exposition to environmental tobacco smoke (ETS) on cough reflex sensitivity (CRS)—concentration of capsaicin causing at least two coughs—in boys and girls (expressed as geometric mean ±95% CI), ^*^p < 0.05.

Multiple comparisons testing have shown that CRS in girls exposed to ETS [22.3 μmol/l (9.8–50.2 μmol/l)] was significantly higher compared to CRS of not exposed girls [79.9 μmol/l (56.4–112.2 μmol/l), *p* = 0.02] as well as compared to exposed boys [121.4 μmol/l (58.2–253.1 μmol/l), *p* = 0.01]. No statistical difference in CRS was seen between girls and boys not exposed to ETS [79.9 μmol/l (56.4–112.2 μmol/l) vs. 64.9 μmol/l (46.5–90.9 μmol/l), *p* = 0.8] and between boys exposed and not exposed to ETS (Figure 1, Table 1).

**Table 1:** **Subjects characteristics, living in urban area and incidence of cough lasting more than 3 weeks in the preceding year in children not exposed (no-ETS) and exposed to environmental tobacco smoke (ETS)**.

	**no-ETS**	**ETS**
No.	247	43
Girls	119	19
Boys	128	24
**HEIGHT (cm)**		
Girls	160.0 (14.2)	150.8 (16.0)^**^
Boys	165.6 (15.4)^§§^	153.4 (17.5)^**^
**WEIGHT (kg)**		
Girls	49.8 (11.7)	44.6 (12.8)
Boys	55.1 (14.6)^§§^	42.7 (15.1)^**^
**FEV1 (% PREDICTED)**		
Girls	91.4 (10.0)	89.6 (15.7)
Boys	96.8 (13.9)^§§^	96.1 (12.6)
**FEV1/FVC (%)**		
Girls	91.5 (7.0)	87.5 (10.0)^*^
Boys	89.7 (8.1)	87.8 (4.7)
**CRS**^#^		
Girls	79.9 (56.4–112.2)	22.3 (9.8–50.2)^*^
Boys	64.9 (46.5–90.9)	121.4 (58.2–253.1)^§§^
**URBAN AREA**		
Girls	81 (68.1)	17 (89.5)
Boys	91 (71.1)	19 (79.2)
**WET COUGH**		
At least 1 episode	73 (29.5)	13 (30.2)
Girls	34 (28.6)	6 (31.6)
Boys	39 (30.5)	7 (29.2)
More than 1 episode	23 (9.3)	4 (9.5)
Girls	9 (7.6)	2 (10.5)
Boys	14 (11.0)	2 (8.7)
**DRY COUGH**		
At least 1 episode	56 (22.7)	15 (34.9)
Girls	26 (21.8)	7 (36.8)
Boys	30 (23.4)	8 (33.3)
More than 1 episode	14 (5.7)	7 (16.7)^**^
Girls	4 (3.4)	4 (22.2)^**^
Boys	10 (7.9)	3 (12.5)

*Between groups comparisons of subjects characteristics performed by two sample T-test. Between groups comparisons of living in urban area and cough incidence provided by χ^2^ test (if n ≥ 5) or Fisher's exact test. Results are presented as mean (SD) or n (%), except where indicated*.

# *mean (95% CI)*.

Significance of difference between two exposition groups of the same gender at the level of

** *p < 0.01*,

* *p < 0.05*.

*Significance of difference between gender of the same exposition group at the level of ^§^p < 0.05*,

§§ *p < 0.01*.

#### Effect of living area

To study individual effect of living area on CRS, analysis was performed in no-ETS group of children (*n* = 247).

**Figure 2:**
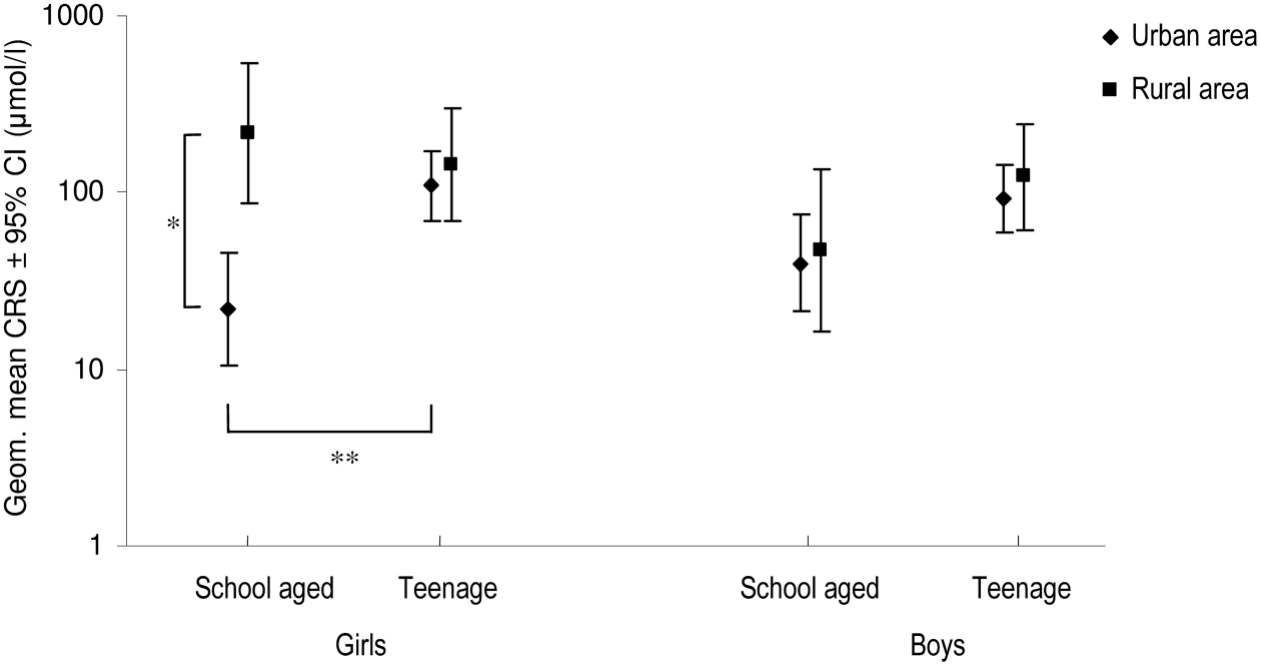
**Effect of living area on cough reflex sensitivity (expressed as geometric mean of CRS ±95% CI) in school aged vs. teenage children not exposed to environmental tobacco smoke**. ^*^Significance of difference between urban and rural school-aged girls at the level of *p* = 0.001. ^**^Significance of difference between school-aged and teenage girls in urban area at the level of *p* = 0.001.

Three factorial ANOVA design between “age group” by “gender” by “living area” has revealed a significant three way interaction [*F*_(1, 220)_ = 4.051, *p* = 0.04]. As can be seen in Figure 2 and Table 2, multiple comparisons testing revealed that the only significant difference in CRS between two living areas was found in group of school aged girls and the only significant difference in CRS between two age groups was found in urban area girls. No significant difference in CRS between two living areas or two age groups was found for boys. Finally, there was no significant difference in CRS between the two genders at any level of age group and living area.

**Table 2:** **Subjects' characteristics and incidence of cough lasting more than 3 weeks in the preceding year in children not exposed to environmental tobacco smoke divided according to age group and living in urban or rural area**.

	**Urban area**		**Rural area**	
	**School age**	**Teen age**	**School aged**	**Teen age**
**NO**.				
Girls	23	58	15	23
Boys	30	61	11	26
**HEIGHT (cm)**				
Girls	144.7 (14.6)	168.0 (7.1)^**^	143.8 (11.6)	165.9 (5.0)^*^
Boys	150.8(9.8)	171.9 (11.8)^**^^§^	147.6 (10.1)	175.4 (10.9)^**^^§§^
**WEIGHT (kg)**				
Girls	38.9 (11.5)	54.7 (8.2)^**^	37.8 (6.9)	55.9 (8.4)^**^
Boys	42.0 (9.0)	60.6 (11.8)^**^^§§^	38.7 (7.4)	64.2 (12.3)^**^^§§^
**FEV1 (%)**				
Girls	94.5 (10.5)	89.3 (9.2)^*^	94.6 (10.9)	91.7 (10.2)
Boys	95.7 (10.3)	96.1 (17.0)^§§^	97.5 (5.2)	99.4 (12.0)®
**FEV1/FVC (%)**				
Girls	91.8 (5.5)	90.6 (7.5)	92.6 (8.4)	92.8 (5.9)
Boys	90.2 (7.4)	89.5 (9.1)	90.1 (6.2)®	89.3 (7.5)
**CRS**^$^				
Girls	22.0 (10.6–45.6)	108.8(68.7–172.9)^*^	215.9(87.3–533.4)^#^	142.7(68.6–296.5)
Boys	40.0 (21.1–75.2)	92.7(59.1–145.0)	47.2 (16.3–135.6)	122.8(61.7–244.2)
**WET COUGH**				
At least 1 episode	19 (35.8)	32 (28.6)	9 (34.6)	11 (22.4)
Girls	7 (30.4)	20 (34.5)	4 (26.7)	3 (13.0)
Boys	12 (40.0)	14 (22.9)	5 (45.4)	8 (30.8)
More than 1 episode	6 (11.3)	12 (10.1)	2 (7.7)	3 (6.2)
Girls	1 (4.3)	6 (10.3)	1 (6.7)	1 (4.3)
Boys	5 (16.7)	6 (9.8)	1 (9.1)	2 (8.0)
**DRY COUGH**				
At least 1 episode	13 (24.5)	27 (22.7)	4 (15.4)	12 (24.5)
Girls	5 (21.7)	13 (22.4)	3 (20.0)	5 (21.7)
Boys	8 (26.7)	14 (22.9)	1 (9.1)	7 (26.9)
More than 1 episode	4 (7.5)	6 (5.1)	1 (3.8)	3 (6.4)
Girls	0 (0.0)	2 (3.4)	1 (6.7)	1 (4.5)
Boys	4 (13.3)	4 (6.7)	0 (0.0)	2 (8.0)

*Between groups comparisons of subjects characteristics performed by two sample T-test*.

Between groups comparisons of cough incidence provided by χ^2^ test (if n ≥ 5) or Fisher's exact test Results are presented as mean (SD) or n (%), except where indicated

$ mean (95% CI)

Significance of difference between two age groups of the same living area group at the level of

** *p < 0.01*,

* p < 0.05

Significance of difference between urban and rural children of the same age at the level of

# p < 0.05

Significance of difference between gender of the same age and living area group at the level of

§ *p < 0.05*.

### Incidence of wet and dry cough lasting more than 3 weeks

As seen in Table 1, the incidence of at least 1 episode of wet cough lasting more than 3 weeks and the incidence of more than 1 episode of wet cough lasting more than 3 weeks, no significant differences were found between no-ETS group when compared to ETS. No difference in incidence of these parameters was seen either between two genders in any of ETS exposure groups, or between no-ETS group when compared to ETS group of the same gender.

On the other hand, the incidence of more than 1 episode of dry cough lasting more than 3 weeks was significantly higher in the ETS compared to no-ETS group. When children were divided according to gender, this significant difference was seen in girls, but not in boys.

No other differences in dry cough parameters were seen between children of no-ETS group when compared to ETS group.

As seen in Table 2, in no-ETS group, no significant difference in wet and dry cough parameters was seen between children living in urban compared to rural area when controlled for age group and gender.

### Dimensional determinants and spirometry parameters

As seen in Table 1, mean height, both for girls and boys, was significantly different between no ETS and ETS group. Mean weight between no ETS and ETS group was significantly different in boys, but not in girls.

Concerning spirometry parameters, FEV1 was not different between two exposition groups when controlled for gender. However, FEV1/FVC was significantly lower in girls exposed to ETS compared to not exposed girls.

A significant difference in height, weight and FEV1 was seen between two genders of children not exposed to ETS. On the other hand, no such differences were seen in the group of children exposed to ETS.

As seen in Table 2, dimensional determinants and spirometry parameters did not significantly differ between urban and rural children not exposed to ETS, when controlled for age group and gender.

A significant difference between two age groups of the same living area was seen for height and weight, both in boys and girls. Concerning spirometry parameters, urban teenage girls had significantly lower FEV1 compared to urban school aged girls. No such difference was seen for urban boys or for rural girls and boys. No significant difference in FEV1/FVC was seen between two age groups of the same living area, both in girls and boys.

## Discussion

The purpose of this study was to find out whether factors of indoor and outdoor air pollution may contribute to age- and gender specific differences in cough outcomes—CRS to capsaicin and incidence of chronic cough. Our results have confirmed previous reports (Varechova et al., 2008; Chang et al., 2011) that CRS in children decreases with age and does not differ between genders, when environmental exposures are not taken into account (see Section Effect of Age Group And Gender). Moreover this study found a new bit of information: increased exposure to ETS due to parental smoking and increased exposure to PM_10_ due to living in urban area make a difference and their effect—increase in CRS—is gender specific, linked to female gender. However, the effects of parental smoking and living area on cough differed in some extent. While the effect of parental smoking on CRS was observed in girls independently of age group, its effect seems to be gender- but not age-related. On the contrary, the effect of living in urban area on CRS was seen only in school aged girls and its effect seems therefore to be age-and gender-related. Altogether, this difference between the effects of parental smoking and living in urban area on CRS suggest that ETS and PM10 may up-regulate cough by different mechanisms, at different levels of cough neural circuitry and/or the degree of exposure to indoor compared to outdoor air pollution may differ according to age and gender.

### Effect of ETS on cough

The mechanisms by which ETS may cause cough have been largely studied using preclinical models. The results obtained thanks to the single-fiber recording technique in anesthetized, artificially ventilated dogs hypothesized that nicotine acting through nicotinic acetylcholine receptors, that are present on variety of cells in the airways, is responsible for cough induced by exposure to cigarette smoke (Lee et al., 2007, 2010; Lee and Gu, 2009). Recent information suggest, that another constituents of cigarette smoke, acrolein and crotonaldehyde, may contribute to cough caused by ETS through their selective activation of calcium channel transient receptor potential ankyrin-1 (TRPA1) ion channel expressed on bronchopulmonary C-fibers. Recent findings show that nicotine is also capable of activating this receptor (Talavera et al., 2009) pointing to the important role of TRPA1 in cough induced by ETS (Grace and Belvisi, 2011). Other pre-clinical studies analysing prolonged exposition to cigarette smoke have shown increased cough response to citric acid or capsaicin in guinea pig *in vivo* (Karlsson et al., 1991; Joad et al., 2004; Lewis et al., 2007). Further, *in vitro* studies in ETS-exposed animals have revealed enhanced peripheral afferent nerve activity, that was dose-dependent (Bonham et al., 1996; Mutoh et al., 1999, 2000) and NTS second order neurone activity (Mutoh et al., 2000). Further, studies of Sekizawa et al. (2008) in young guinea pigs have shown that ETS exposure for the equivalent period of human childhood resulted in an augmentation of evoked synaptic transmission between primary sensory fibers and second order neurons in the NTS by recruiting substance P at the first central synapse. The results of our study together with those reported in preclinical studies strongly suggest the potential of ETS to provoke plasticity changes in neural pathways responsible for regulating cough, leading to central and peripheral sensitization of cough reflex. By this mechanism of action, prolonged exposure to ETS may lead to neuropathic changes at some regulatory moment in the sensorimotor control of cough, as suggested by Chung et al. (2013). This may further lead to development of cough hypersensitivity to otherwise non-tussigenic stimuli and also responsible for increased incidence of chronic cough in exposed subjects. It is speculated here that prolonged exposure to ETS should be considered as a possible cause of cough hypersensitivity syndrome.

On the other hand, our results are in discordance with the results of another clinical study (Wise et al., 2013) that have shown a significantly decreased CRS in children exposed to ETS compared to those not exposed. The discrepancy between the results of two studies might be related to different duration and intensity of ETS exposure. In our study, we focused our attention to current exposure to ETS associated with both parents smoking. Information on ETS exposure was obtained by parent completed questionnaire and we do not have any information about the number of cigarettes smoked per day, nor whether children were exposed to ETS from birth. Therefore, our sample of children exposed to ETS seems to comprise children with ETS exposure of different duration and intensity. On the other hand, Wise et al. studied children of parents that had a high level of nicotine dependence, who smoked about 9 cigarettes per day and all but one child had been exposed to ETS from birth. This led us to suppose that level of children exposure to ETS was higher in the study of Wise and coworkers. It can be argued that exposure to ETS of very high intensity and long duration may result in cough hyposensitivity, similarly as exposure to mainstream tobacco smoke (Dicpinigaitis, 2003; Kanezaki et al., 2012), whereas less intense, acute or prolonged exposure to ETS may result mostly in cough hypersensitivity. In smokers, depression of cough reflex seems to be the result of desensitization of airway cough receptors after chronic exposure to cigarette smoke and is reversed soon after smoking cessation (Sitkauskiene and Dicpinigaitis, 2010). It can be speculated that this mechanism is implied also in decreased cough sensitivity in children of heavy smokers.

As reviewed by Joad et al. (2007), epidemiological studies show that cough—especially chronic dry one- is strongly associated to current and previous ETS exposure in children and adults (Wakefield et al., 2003; Salo et al., 2004; David et al., 2005). However, no direct information exists about stronger effect of ETS on cough outcomes in females compared to males. The results of our study with healthy children suggest that when subjects are controlled for age and gender, the link between cough and exposition to ETS is dependent on gender. Here, not only increased CRS but also a higher incidence of dry chronic cough was observed only in girls. The fact that cough hypersensitivity in our study was found only in females exposed to ETS, that can be explained by enhancement of regional dose of inhaled pollutants in female respiratory tract (see later), supports the presence of dose response relationship between ETS exposure and cough sensitivity. However, more clinical studies focused on this dose response effect of ETS are needed to clarify this concept.

### Effect of PM10 on cough

PM_10_ from rural areas contains mostly non-toxic coarse fraction mainly originating from soil and natural sources, whereas that from urban area largely comprises toxic vehicle-derived transition metals and ultrafine particles, such as diesel soot, that are very likely to mediate adverse health effects of PM_10_(Donaldson et al., 2000). Numerous epidemiological studies suggest that PM_10_ exposure is also strongly associated with cough, and this association seems to be as strong as (Pierse et al., 2006) or even stronger than with wheeze (Bayer-Oglesby et al., 2005). Unlike ETS no direct pre-clinical or clinical studies exist that could provide us with causative factors and possible mechanisms of enhanced cough due to PM_10_. Nonetheless, the components of PM_10_ deposit at different levels of respiratory tract (from upper and lower airways) according to their aerodynamic diameter size, airflow and breathing pattern (Ferin et al., 1992). Therefore the particles with higher aerodynamic diameter, that depose in larynx, trachea and main bronchi may provoke coughing by mechanical stimulation of cough receptors, touch sensitive vagal Aδ-fibers. Ultrafine diesel exhausted particles, on the other side, may provoke cough through stimulation of C-fibers by activation of TRPA1 ion channel (Grace and Belvisi, 2011; Fariss et al., 2013; Shapiro et al., 2013). In addition, ultrafine particles and transition metal components of PM_10_ have been increasingly associated with inflammatory response in airways and lungs (Rastogi et al., 1991; Donaldson et al., 2000; Jiménez et al., 2000; MacNee and Donaldson, 2000); and may result in enhanced coughing due to sensitization of the cough reflex and/or by increased production of mucus. Our results that found increased CRS to capsaicin in school-aged girls living in urban area when compared to rural ones suggest that urban PM_10_ may sensitize cough reflex. On the other side, no difference in chronic cough frequency was found between urban and rural children, even when controlling for age-group and gender. This possibly points to the fact that mean concentration of PM10 in recorded area, that did not often exceed the limits recommended by European Union, was not high enough to produce symptomatic cough in otherwise healthy children.

### Age and gender differences in the effect of air pollution on cough

Our results have shown that both ETS and PM_10_ have the potential to trigger cough and cause cough hypersensitivity. However, the role of age and gender in the effect of air pollution on cough strongly suggest implication of several biological and behavioral factors that change with age and are gender specific. The current concept of central regulation of cough supports the role of higher brain centers, where sensory information related to airway irritation may undergo subcortical (affective) and/or cortical (discriminative) processing giving rise to the respiratory sensation of urge to cough (Davenport, 2009). Urge-to-cough precedes the motor response and thus may allow higher brain centers to activate those efferent inhibitory pathways in situations ranging from basic survival to social embarrassment (Gracely et al., 2007). Recent advances in pain research have pointed slow maturation of endogenous inhibitory descending pathways (Fitzgerald and Walker, 2009; Hathway et al., 2012) that may therefore be less efficient throughout childhood compared to adulthood (La Hausse de Lalouvière et al., 2014; Walker, 2014). As pain and cough share many similarities, it can be speculated that increased central processing of the nociceptive information caused by reduced descending inhibition may result in higher cough outcomes related to environmental pollution during childhood. Moreover, studying functional brain activity in response to capsaicin inhalation suggest that increased central processing of airway irritation stimuli may be also responsible for higher cough outcomes in females compared to males (Morice et al., 2014). In this study, the magnitude of activation of somatosensory cortex by capsaicin inhalation was doubled in females compared to males despite of lower dose of capsaicin used in females. Suggested age and gender differences in central processing of nociceptive information leading to cough could therefore explain, at least partially, increased cough sensitivity in school aged girls living in urban area and in girls exposed to ETS.

Further, striking evidence from epidemiological studies points to the fact that the respiratory health effects of air pollution are much more marked in females on one side (Rumchev et al., 2007) and in children on the other (Heinrich and Slama, 2007; Rumchev et al., 2007; Schüepp and Sly, 2012; Sacks et al., 2014). It is suggested that in females, the increased effect of air pollution on respiratory health outcomes is linked to their biological, hormonal, social or behavioral differences with males (Clougherty, 2010). The reason is that sex linked traits influence biological transport of environmentally derived chemicals while gender determines exposure distribution—where people spend time doing which activities. In females, biological sex mostly determines increased dose and greater deposition of inhaled pollutants (Kim and Hu, 1998), absorption (Jones and Lam, 2006) as well as gas-blood barrier permeability (Bräuner et al., 2009) in respiratory tract. Therefore, one potential modifier of air pollution effect on cough could be size of lungs and airways that remains smaller in females compared to males throughout childhood into the adulthood (Hibbert et al., 1995). This biological factor is suggested to be responsible for an enhancement of regional dose of inhaled pollutants in female respiratory tract and may account to greater cough outcomes and cough sensitization seen in girls participating in our study.

As deposition site and dose of inhaled particles within lungs depends on particle size, breathing pattern and lung structure (Yu et al., 1979; Heyder, 1981), the dose of air pollutants deposited in the airway of children is higher compared to adults exposed to the same concentrations (Moya et al., 2004; Pinkerton and Joad, 2006; Buonanno et al., 2012). The differences in respiratory physiology between children and adults may account for age group-related differences in girls exposed to PM10 in our study. Though, absence of such difference in children exposed to ETS and in boys exposed to PM10 suggests implication of other, possibly behavioral factors. Those mostly predispose females to increased exposure to air pollutants present in home environment as in many societies women spend more time at home than men (Redline and Gold, 1994; Krieger, 2003). This may explain, at least partially, why women suffer elevated respiratory symptoms, more asthma and COPD, especially in developing countries (Qureshi, 1994; Ramírez-Venegas et al., 2006). Proportion of indoor and outdoor activities may be different not only between two genders but change over human life span and society. In general, children spend usually more time outdoors than adults performing more activities that increase ventilation rates resulting in increased exposure to air pollutants of outdoor origin (Moya et al., 2004). However, differences of time spent outdoors may occur with onset of puberty. Here, several studies observed that adolescents spend less time outdoors than school-aged children and teenage girls spend smaller proportion of time doing outdoor activities than teenage boys (Peters et al., 1999; Klinker et al., 2014). We suppose that some interplay between the effects of above mentioned factors might be responsible for gender- and age group- related effect of air pollution on cough found in our study.

### Limitations of the study

One limitation of our study could be attributed to the fact that when analysing the effect of ETS on cough we did not take living area into account. The reason was a small sample size of children living in rural area. It could be therefore argued that a significant effect of ETS on CRS, found in girls, may be partially attributed to the effect of living in urban area. However, the distribution of girls and boys living in urban area is not statistically different between ETS and no-ETS group (Table 1). Moreover, a significant increase in CRS was found in urban girls exposed to ETS compared to those not exposed (results not shown). Another limitation of our study is the fact that urge–to-cough, the time spent outdoors, or ETS and PM10 individual dose were not measured. This could bring more information about possible dose response effect between environmental exposure and cough outcomes, so as about relative importance of increased regional dose and changes in central processing of cough in age- and gender- related differences in cough.

## Conclusions

Exposure to indoor and outdoor pollutants is frequently associated with respiratory symptoms, including cough. The results of our study suggest that age and gender related differences in incidence of cough and CRS, found in many studies, might be, at least partially, ascribed to the effect of environmental pollutants. Here, exposure to ETS was associated with increased CRS and more frequent symptom of chronic cough in girls, but not in boys. Living in urban area was associated with increased CRS only in school-aged girls, without any impact on incidence of chronic cough. According to our results, cough reflex pathway may undergo changes as a result of environmental exposure to ETS and PM10 during childhood and adolescence. However, these changes are age and gender dependent, likely driven by some biological and behavioral factors that determine regional dose of air pollutant as well as by specificities in central processing of cough related to development and gender. More clinical studies controlling for age and gender are needed to clarify what factors are behind age- and gender-related effect of air pollution on cough, whether dose response relationship exist between exposure to air pollutants and change in cough sensitivity and finally, whether cough hypersensitivity is long lasting consequence of exposure to air pollutants or it may be reversed by its diminution or cessation.

## Author contributions

SD, JP, JH, and MT have prepared the project of this study. SD and MT managed preparatory phase of the study. SD and JP performed cough reflex sensitivity testing and spirometry in the schools. LM and TZ assured technical assistance during cough reflex sensitivity testing and spirometry. SD and MA performed data collection and statistics. SD and JH have prepared the draft of manuscript. SD, JP, LM, TZ; MA, JH, and MT completed the work and revised the final manuscript.

### Conflict of interest statement

The authors declare that the research was conducted in the absence of any commercial or financial relationships that could be construed as a potential conflict of interest.
